# Evaluation of *Theobroma cacao* L. as a bioindicator for cadmium contamination through H_2_O_2_ electrochemical analysis

**DOI:** 10.1038/s41598-025-11715-2

**Published:** 2025-07-23

**Authors:** Lenys Fernández, Patricio Javier Espinoza-Montero, María José Gallegos-Lovato, Paulo Bustamante, Diego Bolaños-Méndez, Juan Diego Sampedro, Augusto Rodríguez, Andrea Ortega-Gallegos, Mónica Jadan

**Affiliations:** 1 Escuela de Ciencias Químicas, Pontificia Universidad Católica del Ecuador, Quito, Ecuador; 2 Instituto de Investigaciones Fisicoquímicas Teóricas y Aplicadas (INIFTA), La Plata, Argentina; 3 Universidad de las Fuerzas Armadas ESPE, Sangolquí, Ecuador

**Keywords:** *Sedentary bioindicators*, *Theobroma cacao*, *Cadmium contamination*, *Oxidative stress*, *Chronoamperometry*, Environmental sciences, Chemistry

## Abstract

The use of sedentary bioindicators, such as trees, in environmental contamination monitoring is receiving increased focus. This study evaluates *Theobroma cacao* L. as a bioindicator for cadmium (Cd) contamination by quantifying hydrogen peroxide (H₂O₂) as an oxidative stress marker in cellular suspensions exposed to CdSO₄. Chronoamperometric measurements using platinum electrodes indicated Cd accumulation in *T. cacao* L. and revealed a corresponding increase in H₂O₂ production up to a threshold level, beyond which cell apoptosis occurred. These findings support the potential of *T. cacao* L. as a bioindicator of Cd pollution. Moreover, H₂O₂ quantification via chronoamperometry demonstrated a rapid and effective method for detecting Cd-induced oxidative stress in plant systems. Future research should explore field applications, evaluate alternative plant species, and assess long-term responses under real environmental conditions to optimize this approach for large-scale biomonitoring.

## Introduction

Environmental biomonitoring is a crucial tool for assessing contamination risks and their impact on ecosystems. Among the various biomonitoring strategies, sedentary bioindicators—organisms that accumulate and reflect environmental contaminants—are especially useful for evaluating pollution levels over time^1^. Plants in particular are widely used to monitor toxic metal contamination due to their ability to absorb and store hazardous elements from soil and water^2,3^.

Ecuador has a strong agricultural and economic interest in cultivating cacao, or *Theobroma cacao* L. The country’s fine aroma cacao is particularly highly valued in European markets for its distinct flavor and quality^4,5^. While bioindicators are often associated with organisms like lichens and mollusks, which passively reflect environmental contamination, certain plant species can actively serve as effective localized biomonitors of soil pollution. *T. cacao* L., known for its ability to bioaccumulate cadmium (Cd), offers valuable insights into toxic metal contamination in agricultural ecosystems. This is especially relevant in Ecuador, where Cd pollution stems from both natural and anthropogenic sources, including industrial discharges, vehicular emissions, and artisanal mining^11^. Unlike traditional bioindicators, which are widely distributed across different environments, *T. cacao* L. is cultivated in specific agricultural regions, making it a particularly relevant indicator of pollution in economically important crops. Monitoring oxidative stress responses in cacao plants can provide an early warning of Cd exposure, facilitating the evaluation of soil contamination levels and potential risks to food safety. This localized approach to biomonitoring is crucial in regions where toxic metal accumulation threatens both agricultural productivity and compliance with international trade regulations.

Cadmium exposure severely affects plant morphology, causing stunted growth, chlorosis, and impaired photosynthesis, as well as damaging cellular structures such as membranes and DNA^6–8^. One of the primary physiological responses to Cd stress is oxidative stress, which triggers excessive production of reactive oxygen species (ROS), including superoxide anions (O₂^•−^), hydroxyl radicals (^•^OH), and hydrogen peroxide (H₂O₂)^9^. Notably, Cd exposure has been shown to significantly increase H₂O₂ levels in plant systems, making it a useful marker for oxidative stress.

Electrochemical techniques offer sensitive and rapid methods to detect ROS, providing advantages over traditional spectrophotometric and chromatographic approaches^3,4^. In particular, chronoamperometry allows real-time monitoring of H₂O₂ production in plant cell suspensions, making it a reliable indicator of Cd-induced oxidative stress^10^.

Considering these factors, this study aimed to explore the potential of *T. cacao L.* as a sedentary bioindicator by quantifying H_2_O_2_ in cellular suspensions derived from seed explants exposed to Cd^2+^ ions. The suspensions were cultivated under controlled in vitro conditions utilizing a medium simulating the optimal environment for plant tissue development^11^. While previous studies have investigated Cd accumulation in cacao-growing soils^16,17^research on the use of cacao as an active bioindicator remains limited^12,13^. This study seeks to bridge that gap by exploring H₂O₂ quantification as a novel approach for assessing toxic metal contamination. The findings will contribute to understanding the role of cacao as an environmental sentinel and demonstrate the applicability of electrochemical techniques in the biomonitoring of toxic metal pollution. Such a methodology could provide a practical alternative for environmental monitoring, particularly in regions where conventional assessment methods are impractical or imprecise.

## Materials and methods

### Reagents

The following reagents were used in this study: calcium nitrate (PhytoTechnology Laboratories, analytical grade); potassium sulfate (Merk, analytical grade); Bacto agar (Biomark); Murashige & Skoog basal medium with vitamins (MS); 2,4-dichlorophenoxyacetic acid (2,4-D); 6-benzylaminopurine (6-BAP); dipotassium phosphate (99%, Merck, analytical grade); monopotassium phosphate (99.05%, Merck, analytical grade); H_2_O_2_ (30% v/v, Sigma-Aldrich), and sulfuric acid (Fischer, analytical grade). An electrode polishing kit (CH Instruments, Inc.) was also employed.

### Equipment

Experiments were conducted using a laminar flow cabinet (Esco); autoclave (Tuttnauer); Olympus BX-41 microscope (Olympus Corporation); orbital shaker (WiseSheak); ultrasonic bath (Branson 3800); and a Neubauer chamber (AR Biotech). Electrochemical measurements were performed with a potentiostat (Biologic SP-150); ECLab software V11.26; platinum (Pt) working electrode; Ag/AgCl reference electrode (3 mol L^−1^ KCl); and graphite rod counter electrode.

### Preparation of *T. cacao* L. cell suspensions

*T. cacao* L. pods were collected in the town of Mindo, Ecuador, (latitude: −0.0506,

longitude: −78.7788) in the province of Pichincha. The fruit was transported to the laboratory in a portable refrigerator. The disinfection process involved extracting and rinsing the *T. cacao* L. seeds with distilled water to remove the mucilage completely, followed by washing with a chlorine solution, testing concentrations between 3.5 and 5% for 10 min. The seeds were then rinsed three times with distilled water inside a laminar flow chamber^14^.

Callus formation was induced using MS medium containing hydrated calcium nitrate (1967 mg L^−1^), potassium sulfate (1559 mg L^−1^), sucrose (40 g L^−1^), 6-BAP (4 mg L^−1^), 2,4-D (2 mg L^−1^), and 7 g of agar^15^.

Subsequently, four explants per flask were planted in MS medium and stored at room temperature under dark conditions for 30 days to allow for callus multiplication^14^. The same culture medium was used for both multiplication of callus and sowing.

To prepare the cellular suspensions, approximately 1 g of the formed callus was added to 50 mL of the aforementioned MS growing medium (excluding the agar). The mixture then underwent orbital agitation^15^.

Cells were counted by taking a sample of the suspension inside the flow chamber, which was subsequently introduced into the Neubauer chamber. The cells were observed and counted using an Olympus BX-41 microscope. Cellular concentration (CC) was calculated using Eq. (1):

1 $$\:CC=\frac{\#\:viable\:cells}{\#\:squares}*\frac{64\:squares}{1\:square}*\frac{1\:square}{\left(length*height*depth\right){mm}^{3}}*\frac{1000\:{mm}^{3}}{1\:{cm}^{3}}$$

### Hydrogen peroxide production assessment

The electrochemical system consisted of a Pt working electrode, a graphite rod counter electrode, and a Ag/AgCl (3 mol L^−1^ KCl) reference electrode. The Pt working electrode was cleaned using successive mechanical polishing with alumina powder of decreasing grain sizes (1, 0.3, and 0.05 μm)^16^ for 3 min for each grain size. Subsequently, electrochemical cleaning was performed via cyclic voltammetry in a 0.5 mol L^−1^ H_2_SO_4_ solution, with potentials ranging from −0.300 to 1.800 V *vs.* Ag/AgCl at a scan rate of 100 mV s^−1^.

Chronoamperometric measurements were performed in cell suspensions with a turbidity of 15 NTU and CC of 3 × 10^4^ cells mL^−1^. Turbidity of initial cell solution 25 NTU and the CC of the stock solution was of 5 × 10⁴ cells mL^−1^.

Calibration curves were constructed by measuring chronoamperometric currents in H_2_O_2_ standard solutions at concentrations ranging from 0.3 to 1.4 µmol L^−1^ in a phosphate buffer solution (PBS) at pH 5.7. The standard addition method was employed to determine H_2_O_2_ concentrations to minimize the dependency between the target signal current and possible sample matrix interference^26^. Method performance parameters, including sensitivity, detection limit, quantification limit, precision, and accuracy, were evaluated. The arithmetic mean, standard deviation, coefficient of variation, and percent recovery (R%) were also calculated for the measurements (*n* = 5). The percent coefficient of variation (CV%) values were assessed according to the acceptable limits described in the AOAC (2012) guidelines for laboratory chemical method validation in dietary supplements and botanicals. These were set at CV < 11% for reproducibility and precision and between 80 and 115% for R% values.

For the respective cellular stress experiments, 1:10 dilutions of aqueous suspensions were prepared using CdSO_4_ concentrations of 5, 20, 50, and 100 µmol L^−1 4^. Cells were exposed to the stressing agent for 1, 2, 3, 4, 5, and 6 h. H_2_O_2_ quantification was performed using the standard addition method in a 0.1 mol L^−1^ PBS at pH 5.7. Aliquots of 20 µL were added from a 0.02 mol L^−1^ H_2_O_2_ standard solution every 20 s^16,17^.

## Results and discussion

### Disinfection protocol for *T. cacao* L. explants

The most efficient disinfection protocol for *T. cacao* L. explants was determined by evaluating bacterial contamination, fungal contamination, and oxidation. The optimal method involved immersing the cacao seeds, with mucilage removed, in a 3.5% chlorine solution for 10 min (Table 1), followed by three rinses with distilled water in a laminar flow chamber. This approach minimized oxidation while effectively reducing microbial contamination, preventing inhibition of seed germination. This protocol aligns with reported plant tissue disinfection methods, where appropriate NaClO concentrations have been shown to reduce contamination without significantly affecting germination or development^18^. The selection of a 3.5% chlorine concentration effectively balanced microbial decontamination while maintaining explant viability, ensuring a suitable starting point for subsequent experimental procedures.

**Table 1:** Results of analyzed variables for determining optimal disinfection protocol.

	Chlorine concentration (%)	
**Evaluated variables**	3.5	5.0
**Fungal contamination**	10	12
**Bacterial contamination**	3.0	5
**Oxidation**	5.0	17

### Induction and multiplication of embryogenic callus

**Fig. 1:**
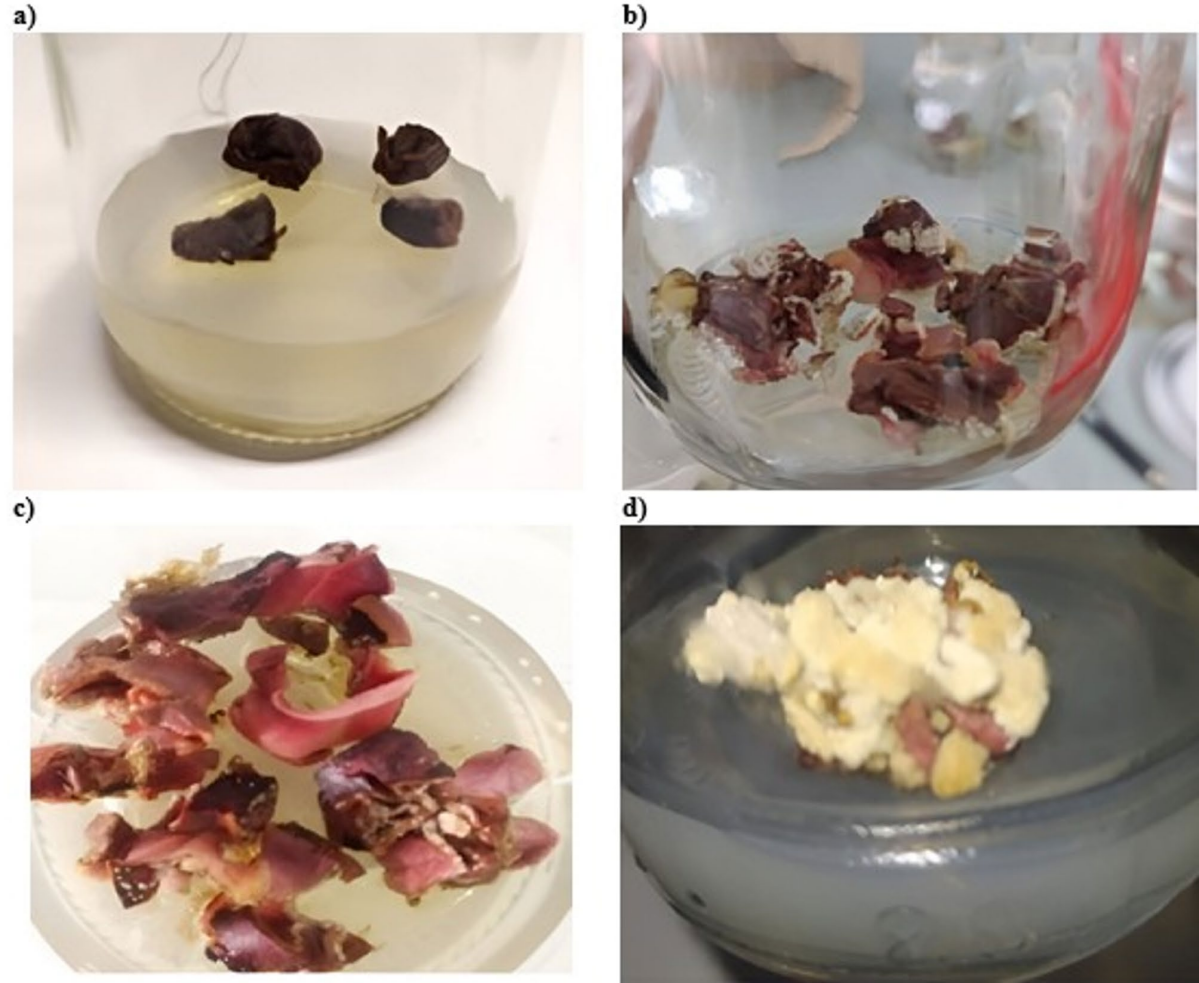
*Theobroma cacao* L. seeds (**a**) immediately after sowing in the medium; (**b**), (**c**) callus formation 15 days post-sowing; (**d**) callus after 30 days.

Embryogenic callus formation in *T. cacao* L. seeds began seven days after sowing in MS medium. The calluses were kept in a dark environment to promote optimal growth. After 15 days, their successful development confirmed that the MS medium provided suitable conditions for explant cultivation (Fig. 1). After 30 days, an additional multiplication step was carried out using the same MS medium. Figure 1d shows the embryogenic callus alongside clusters of undifferentiated cells. The MS medium, enriched with macro- and micronutrients, vitamins, and cytokinins, created optimal circumstances for callus growth under in vitro conditions.

The induction and multiplication of embryogenic callus in *T. cacao* L. are critical steps in somatic embryogenesis and plant regeneration protocols. In this study, embryogenic callus formation began seven days after sowing in MS medium under dark conditions, consistent with established methodologies. Subsequent sub-culturing after one month further promoted callus proliferation.

The enriched MS medium provides essential support for in vitro plant tissue culture. The inclusion of plant growth regulators, such as 2,4-D, a synthetic auxin, and 6-BAP, a cytokinin, plays a key role in callus induction and somatic embryogenesis. Research has demonstrated that 2,4-D promotes cell division and dedifferentiation, while 6-BAP enhances cell proliferation and differentiation. Their synergistic effect has been shown to facilitate embryogenic callus development in cacao^19^.

Regular sub-culturing onto fresh medium is essential for maintaining cell viability, preventing necrosis, and ensuring continued callus proliferation. This practice replenishes nutrients and growth regulators while eliminating inhibitory metabolites. Studies have confirmed that sub-culturing enhances callus growth and embryogenic potential in cacao tissue cultures^19^.

Our results are consistent with those of previous studies highlighting the positive influence of 2,4-D and 6-BAP supplementation on callus development^20^. To prevent cell death and aggregation, subcultures were periodically transferred to fresh medium^22^. Auxins such as 2,4-D are frequently used to induce embryogenic callus, as they regulate key physiological and molecular processes involved in somatic embryogenesis. Consistent with our observations, Hazubska-Przybył et al.^27^ reported that the combination of 2,4-D and 6-BAP enhances somatic embryo formation from embryogenic callus structures.

### Cell counting

**Fig. 2:**
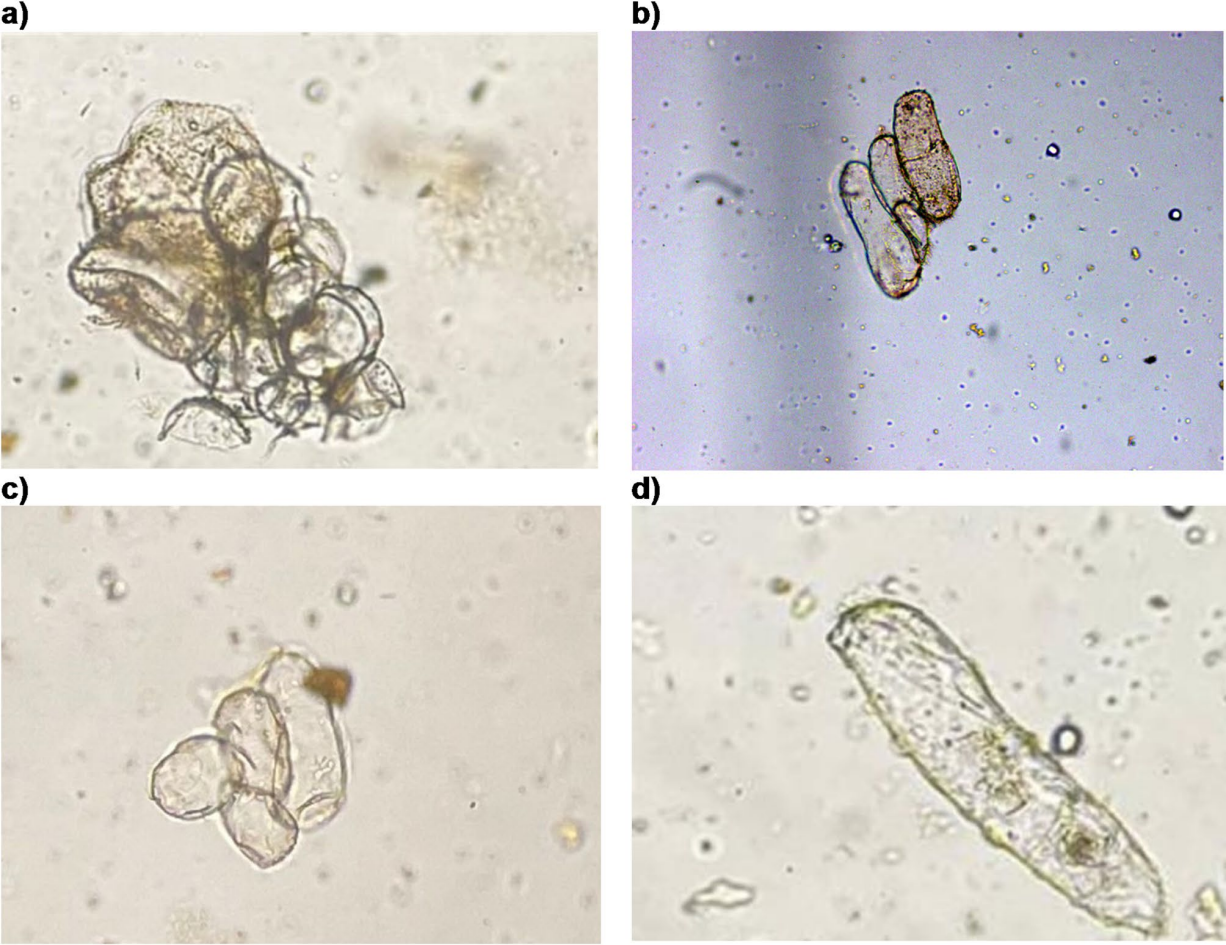
Microscopic images (10x magnification) of *Theobroma cacao* L. cell suspensions: (**a**–**c**) fully dispersed cells and (**d**) smaller cell clusters.

Cell counting was conducted by taking a small aliquot from each suspension, placing it in the Neubauer chamber, and examining it under a microscope. Figure 2 shows optical microscope images of the cells at 10x magnification, showing fully disaggregated cells, confirming the suspensions were suitable for monitoring cell growth. Additionally, no large clusters of cells were observed.

### Evaluation of analysis method

**Fig. 3:**
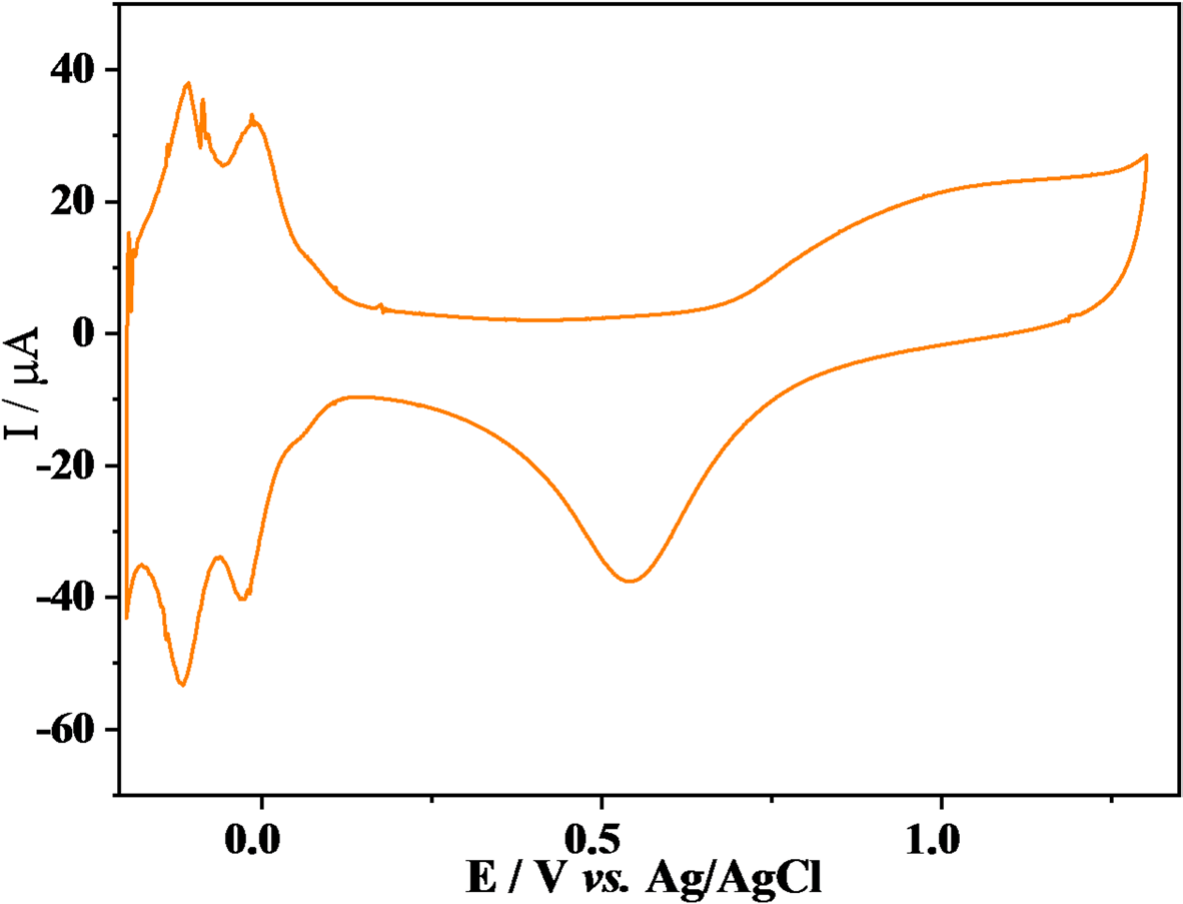
Cyclic voltammogram of the Pt electrode in 0.5 mol L^−1^ H_2_SO_4_ at a scan rate of 100 mV s^−1^, illustrating the electrochemical cleaning process.

Figure 3 shows the cyclic voltammetry responses recorded over 10 consecutives cycles of the electrochemical cleaning process. As described in the experimental section, the working electrode was rinsed before each measurement to prevent analyte interference that could lead to adsorption on the electrode surface; such adsorption may introduce impurities and result in non-stable electrochemical responses^23^. The voltammograms exhibit characteristic peaks corresponding to a H_2_SO_4_ solution at a pure poly-crystalline Pt surface^24^.

According to Strandberg et al.^10^the voltammogram in Fig. 3 can be divided into four regions: (i) the hydrogen adsorption/desorption region ($\:Pt+{H}^{+}+{e}^{-}\rightleftarrows\:Pt-\:{H}_{ads}$) between 0.00 and −0.2 *vs.* Ag/AgCl; (ii) the double layer region between 0.1 and 0.4 *vs. *Ag/AgCl; (iii) the oxidation region during the anodic scan from 0.7* vs.* Ag/AgCl up to the upper potential limit; and (iv) the reduction region during the cathodic scan from at an upper potential limit down to 0.6 Ag/AgCl.

**Fig. 4:**
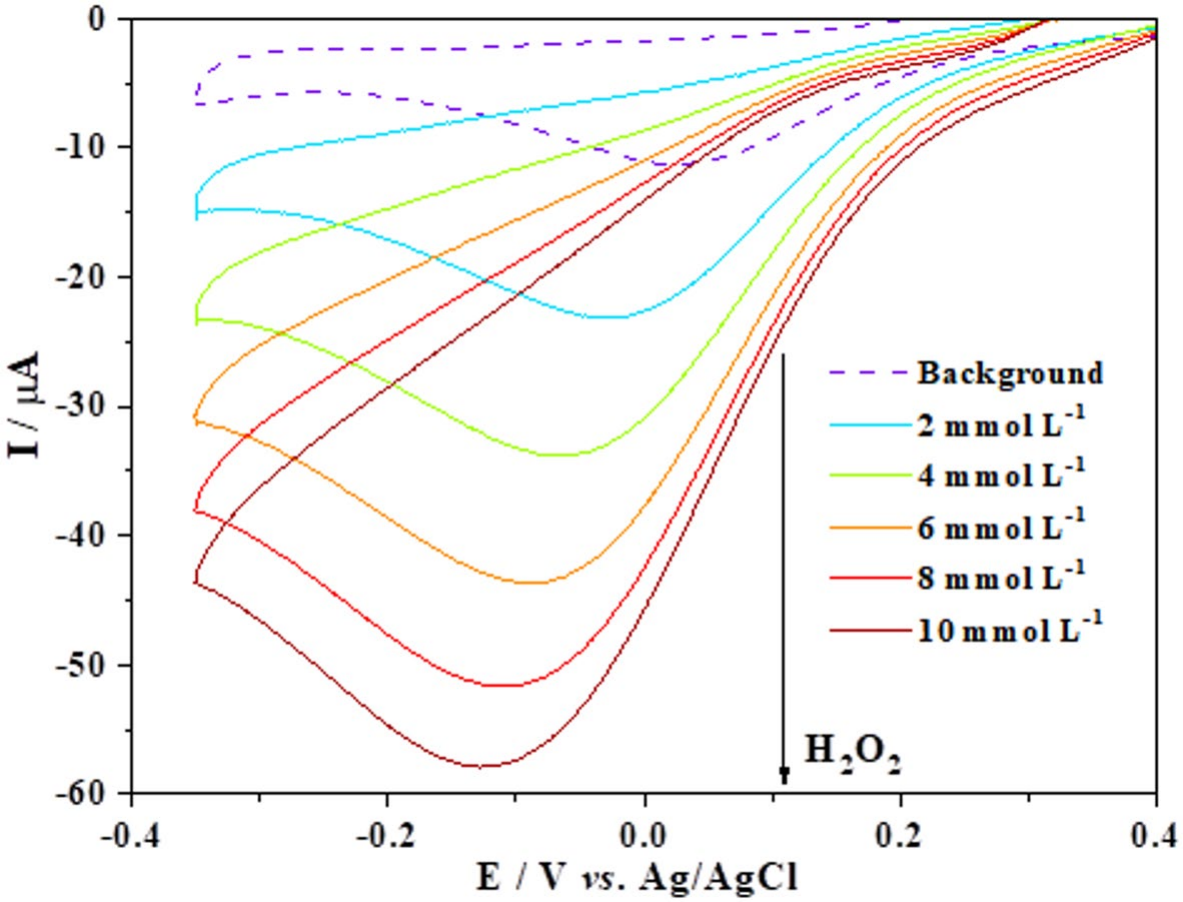
Cyclic voltammograms of the Pt electrode in phosphate buffer solution (pH 5.7) at a scan rate of 100 mV s^−1^, recorded with increasing H_2_O_2_ concentrations.

To assess the electrocatalytic activity of Pt in the H_2_O_2_ reaction, cyclic voltammograms were recorded in a 0.1 mol L^−1^ PBS saturated with N_2_ (Fig. 4). When the Pt electrode was cycled within a potential window ranging from 0.7 V to −0.5 V *vs.* Ag/AgCl, a distinct redox peak was observed at 0.25 V, corresponding to the H_2_O_2_ reduction reaction. The current magnitude increased proportionally with H_2_O_2_ concentration, confirming the catalytic activity of the Pt electrode toward H_2_O_2_ reduction, consistent with prior studies^25^.

**Fig. 5:**
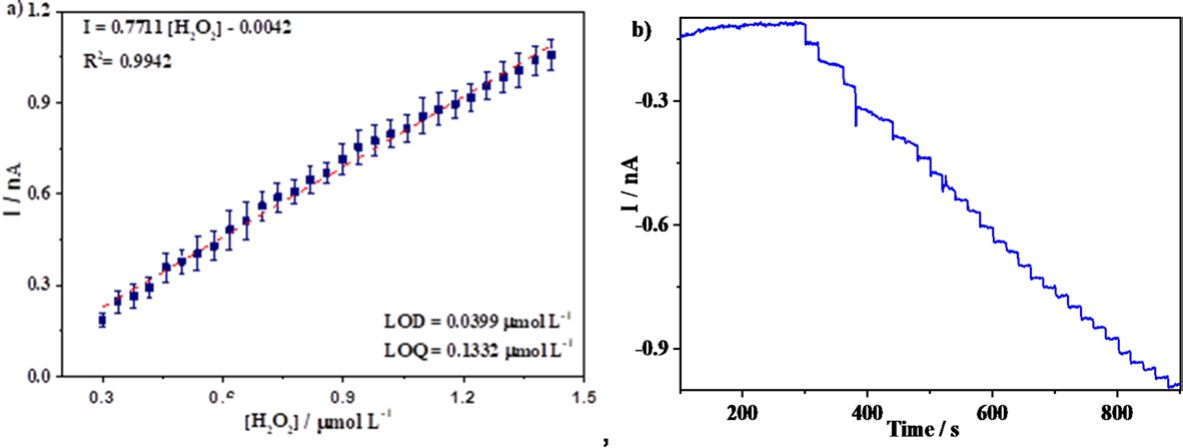
(**a**) Calibration plot for H_2_O_2_ detection on the Pt electrode (3 mm diameter). (**b**) Chronoamperogram.

The calibration plot was constructed using chronoamperometry at a potential of −0.3 V *vs. *Ag/AgCl over a 15 minute period. Figure 5 shows the resulting calibration curve, with the insert displaying the chronoamperogram indicating the electrocatalytic response of the Pt electrode across varying H_2_O_2_ concentrations (0.3–1.4 µmol L^−1^). The maximum reduction currents corresponding to each H_2_O_2_ concentration increased as the potential was adjusted to −0.3 V. However, saturation of the sensor was observed after the addition of 1.4 µmol L^−1^ H_2_O_2_. The limits of detention and quantification were calculated using the formula 3 × SD_blank_/slope of the calibration curve and 10 × SD_blank_ from the blank/slope of the calibration curve, respectively (Table 2). The analytical sensitivity was determined to be 0.4959 mA (µmol L^−1^)^−1^.

**Table 2:** Limits of detection, quantification, and coefficient of variation based on the repeatability and reproducibility of the method developed to quantify H_2_O_2_.

Limit of detection (µmol L^−1^)	Limit of quantification (µmol L^−1^)	Coefficient of variation (%)	Recovery (*R*%)	Parameter
0.012 ± 0.041	0.388 ± 0.001	3.14 ± 0.01	95. 73 ± 0.034	Repeatability
0.013 ± 0.043	0.924 ± 0.003	2.94 ± 0.03	99.44 ± 0.024	Reproducibility
0.012 ± 0.050	0.377 ± 0.004	3.17 ± 0.05	105.35 ± 0.014	
0.013 ± 0.020	0.386 ± 0.003	3.45 ± 0.03	98.38 ± 0.017	Mean

### Hydrogen peroxide quantification in cell suspensions

**Fig. 6:**
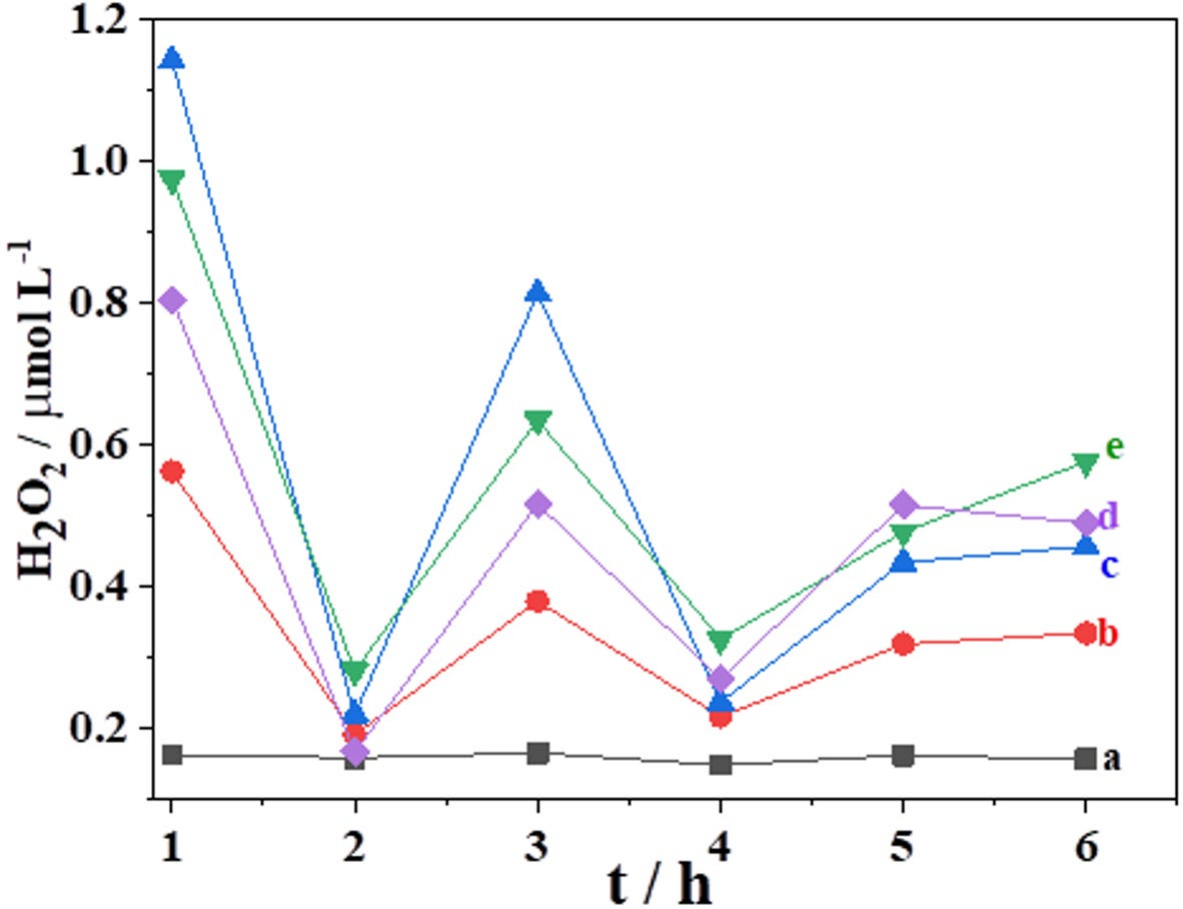
Relationship between CdSO_4_ exposure time and H_2_O_2_ concentration: (**a**) 0 µmol L^−1^ CdSO_4_; (**b**) 5 µmol L^−1^ CdSO_4_; (**c**) 20 µmol L^−1^ CdSO_4_; (**d**) 50 µmol L^−1^ CdSO_4_; and (**e**) 100 µmol L^−1^ CdSO_4_.

While ROS are also metabolic by-products in processes such as photosynthesis, respiration, and nitrogen fixation in plants, their concentrations are normally regulated by scavenging agents, including superoxide dismutase, catalase, glutathione reductase, and peroxidase. Thus, living organisms can maintain harmless levels of ROS concentrations during these processes. However, under stress conditions, this balance is disrupted, resulting in the overproduction of ROS and a subsequent oxidative burst a critical and rapid detoxification response in plants^10,27,28^. Previous studies have indicated that a H_2_O_2_ concentration of just 2.5 mmol L^−1^ in the medium can initiate a series of chain reactions that irreversibly damage DNA, thereby affecting cells’ replication mechanisms^29^. Conversely, at concentrations exceeding 2.5 mmol L^−1^, H_2_O_2_ can damage enzymatic cellular compounds containing Ca^2+^^30^ and attack proteins and lipids within cell membranes, causing lysis^31^. The control experiment, in which the suspension was not exposed to CdSO_4_, was conducted in PBS at pH 5.7, resulting in a mean H_2_O_2_ concentration of 0.16 µmol L^−1^ (Fig. 6a). Figure 6b–e illustrates the relationship between generated H_2_O_2_ concentrations and exposure time in *T. cacao* L. cells subjected to different concentrations of CdSO_4_. There was no change in H_2_O_2_ concentration in the control medium (Fig. 6a) over 6 h, suggesting that, in the absence of oxidative stress, natural ROS scavenging agents effectively maintain H_2_O_2_ concentration at a constant level.

After 1 h of the cell suspension’s exposure to CdSO_4_ solutions, the quantified H_2_O_2_ at the electrode was considerably higher than that in the unstressed suspension (Fig. 6b–e). Thus, 1 h was determined to be the minimum time required to detect H_2_O_2_ in the system due to the oxidative stress process. As shown in Fig. 6, the cell suspension’s response to increasing CdSO_4_ concentrations is consistent with previously reported data^32^; specifically, increasing the amount of Cd^2+^ in the medium led to higher H_2_O_2_ production by cells. The suspension exposed to 5 µmol L^−1^ CdSO_4_ (Fig. 6b) exhibited the lowest H_2_O_2_ concentration over time, while the highest concentration corresponded to the suspension exposed to 100 µmol L^−1^ CdSO_4_ (Fig. 6c). In all cases, peak H_2_O_2_ production was quantified at 3 h, regardless of CdSO_4_ concentration. Overall, Fig. 6 shows that after 1 h of exposure to different CdSO_4_ concentrations, H_2_O_2_ concentrations are higher than those in the control medium, supporting the occurrence of an oxidative burst.

According to the graph shown in Fig. 6, the highest H_2_O_2_ concentrations after adding CdSO_4_ to the cell suspensions occur at 1, 3, and 5 h. Each peak in H_2_O_2_ concentration can indicate an oxidative burst at the respective CdSO_4_ concentration. Conversely, the minimum H_2_O_2_ concentrations at 2 and 4 h likely correspond to periods wherein the cells gradually recover equilibrium, as these concentrations are the same as or very close to those for the control, 0.16 µmol L^−1^ H_2_O_2_ (Fig. 6a).

The results suggest that the overproduction of H_2_O_2_ in cell suspensions of *T. cacao* L. seeds was related to the plant’s defense mechanism against Cd^2+^ ions. Upon exposure to the metal, cell surface receptors recognize it, stimulating localized H_2_O_2_ production. H_2_O_2_ delays germination, allowing cells more time to activate their defense mechanisms, which contributes to resistance to Cd^2+^ stress. However, cell suspension behavior after 5 h of CdSO_4_ exposure suggests that *T. cacao* L. seed cells lose their capacity to re-establish equilibrium H_2_O_2_ concentrations beyond this duration. This is evidenced by the increased production of H_2_O_2_ in all suspensions, regardless of CdSO_4_ concentration, which fails to return to the baseline H_2_O_2_ concentration observed in the control. Notably, cell suspensions exposed to 100 µmol L^−1^ CdSO_4_ (Fig. 6c) showed the lowest capacity for recovery.

The addition of H_2_O_2_ is a commonly used method to induce cellular oxidative stress, typically requiring H_2_O_2_ concentrations greater than 100 µmol L^−1^^33,34^. According to Fig. 6, after 5 h of exposure, cell apoptosis could occur due to the overproduction of H_2_O_2_ caused by stress from the contaminating metal, thereby impairing the cells’ ability to recover. This reaction produces molecular oxygen through the catalase reaction, a very rapid reaction that drastically decreases H_2_O_2_ concentration within minutes^35^. Additionally, the results highlight the efficacy of intracellular antioxidant defenses against H_2_O_2_.

These findings support the potential of *T. cacao* L. as a bioindicator for Cd contamination in the environment. The observed correlation between Cd exposure and H₂O₂ production suggests that oxidative stress responses in cacao cells can serve as an early warning system for toxic metal pollution. The plant’s ability to accumulate Cd and generate a measurable oxidative stress response highlights its suitability for biomonitoring applications, particularly in regions where cacao cultivation is economically and environmentally important. Furthermore, the progressive loss of cellular recovery capacity under prolonged Cd exposure underscores the potential long-term impacts of toxic metal contamination on cacao plantations.

Integrating electrochemical H₂O₂ quantification into environmental monitoring programs could provide a rapid and sensitive method for detecting metal-induced oxidative stress, reinforcing the role of *T. cacao* L. as a practical and sustainable bioindicator for assessing environmental Cd contamination. Additionally, recent studies have highlighted the critical function of phenolic compounds in plant defense mechanisms against Cd stress. These non-enzymatic antioxidants act as ROS scavengers and metal chelators, thereby mitigating oxidative damage and enhancing stress tolerance^42^. *T. cacao* L. phenolic compounds may play a crucial role in maintaining redox balance under Cd stress, complementing enzymatic defenses. Future studies exploring this interaction could further strengthen the potential of *T. cacao* L. as a biomonitor for Cd contamination.

## Conclusion

The determination of H₂O₂ via chronoamperometry using Pt as the working electrode proved to be an effective methodology for Cd quantification, achieving detection and quantification limits of 0.012 and 0.389 µmol L^−1^, respectively. The findings showed that *T. cacao* L. responds to Cd stress through increased H₂O₂ production, demonstrating its potential as a sedentary bioindicator for environmental contamination. The observed correlation between Cd levels and oxidative stress marker suggests that *T. cacao* L. could serve as an efficient tool for monitoring toxic metal pollution in agricultural regions. Additionally, the results highlight the effectiveness of the plant’s defense mechanisms, as evidenced by its immediate oxidative response to Cd-induced stress.

To establish *T. cacao* L. as a bioindicator, field-based studies should be conducted to validate laboratory findings under real environmental conditions. Standardized protocols for H₂O₂ quantification in cacao tissues should be developed to ensure reliable and reproducible results. Moreover, policymakers should consider incorporating *T. cacao* L. into environmental monitoring programs, particularly in regions where cacao cultivation is a major economic activity. Regulatory agencies could leverage these findings to establish early warning systems for Cd contamination, helping to safeguard both agricultural sustainability and public health.

## Data Availability

Data “available on request”: lmfernandez@puce.edu.ec.
